# Maternal Third Delay and Associated Factors among Women Admitted for Emergency Obstetric Care in Public Hospitals in Sidama Regional State, Ethiopia

**DOI:** 10.1155/2023/7767208

**Published:** 2023-04-13

**Authors:** Esuyawkal Mislu, Ali Seid, Nigus Bililign, Terefe Woyo, Dubale Dulla

**Affiliations:** ^1^Department of Midwifery, School of Midwifery, College of Health Science, Woldia University, Ethiopia; ^2^Basic Health Science, Dessie Health Science College, Dessie, Ethiopia; ^3^Department of Midwifery, College of Medicine and Health Science, Hawassa University, Ethiopia

## Abstract

**Background:**

Timing to get obstetric care is critical in preventing maternal death and disability. Maternal third delay, the delay in receiving care after reaching health facilities, involves factors related to organization, quality of care, patient referral, and availability of staff and equipment. However, data is limited on maternal third delay and its associated factors at higher health facilities in Ethiopia.

**Objective:**

This study is aimed at assessing the magnitude of maternal third delay and associated factors among women admitted for emergency obstetric care in public hospitals in Sidama Regional State, Ethiopia, 2021.

**Methods:**

An institution-based cross-sectional study was conducted from September to November 2021. Face-to-face interview with a structured questionnaire and data extraction from medical charts were carried out in selected 542 women (using systematic sampling method). The collected data were coded and entered using EpiData, and bivariable and multivariable logistic regression analyses were done using SPSS version 25. Statistical significances were declared at *p* value less than 0.05.

**Results:**

Maternal third delay was identified among 29.3% (95%CI = 25.2 − 33.5) of the respondents. Additionally, women who arrived with a referral from other health facilities (AOR = 0.311, 95%CI = 0.181 − 0.534), well prepared for birth and its complications (AOR = 2.418, 95%CI = 1.51 − 3.869), self-employed (AOR = 0.223, 95%CI = 0.122 − 0.409), being a government employee (AOR = 0.157, 95%CI = 0.063 − 0.396), having ANC follow-up (AOR = 2.795, 95%CI = 1.318 − 5.928), and absence of health professional (AOR = 4.63, 95%CI = 2.857 − 7.50) were significantly associated with maternal third delay.

**Conclusion:**

This study identified that maternal third delay was high, which indicates that women have not received emergency obstetric care in the recommended time range after they arrived at the health facilities.

## 1. Introduction

Timing to receive emergency obstetric care is critical in preventing maternal disability and death. The three delays model, which was developed by Thaddeus and Maine in 1994, enables us to identify indirect factors that contribute to maternal death. This model identifies three critical phases: first delay, delay in the decision to seek care; second delay, delay in identifying and reaching the health facility; and third delay, delay in receiving care at the health facilities [1].

Maternal third delay includes drugs and equipment, policies and guidelines, human resources, facility infrastructure, patient-related, and referral-related [2, 3]. The contribution of maternal third delay to institutional maternal death and disability either alone or in combination with other phases of delays was high [4, 5]. Addressing this type of delay, which involves factors within the health facility, is essential for ensuring that obstetric emergencies are efficiently managed [6, 7].

Currently, there is a paradigm shift from the first and second phase delays to the third phase delay as the main contributor to maternal mortality [4]. In Ethiopia, the Maternal Death Surveillance Report (MDSR) analysis showed that delay three was cited in 48.6% of the total report of maternal deaths which was higher than the previous year's report [8]. Different factors to receive care in health facility impact not only the outcomes at the facility level but also in the community by providing feedback for subsequent decision-making to utilize emergency obstetric care [2].

Women who need emergency obstetric care experience extremely long delays after reaching a health facility [9]. A multicountry study done in seven countries (Ethiopia, India, Indonesia, Nigeria, Tanzania, Uganda, and Nepal) found that there was a high level of care seeking for maternal complications in the community. Conversely, there was a significant gap around the provision of maternity services, which needs urgent action to improve it at facility level [10].

Different studies identified that inadequate infrastructure and supplies including bed drug supplies, insufficient health personnel [11, 12], refusal to get treatment, limited ability to pay for different costs despite national policy to provide free service [13, 14], unprofessional behavior of health care providers, and weak coordination of referrals were barriers to maternal health service utilization that results in delay in receiving emergency obstetric care [11]. In Ethiopia, previous studies showed that the magnitude of maternal third delay was 32.6% in Nigist Eleni Mohammed Memorial General Hospital [15], 26.9% in three referral hospitals (Felege Hiywot, Debre Markos, and Debre Birhan Referral Hospital) of Amhara Regional State [16], and 74.7% in two referral hospitals in Addis Ababa [17]. Since there was limited data about the magnitude of maternal third delay and its associated factors in higher health facilities of Ethiopia, this study was aimed at assessing it in public general and specialized hospitals in Sidama Regional State.

## 2. Method and Materials

### 2.1. Study Setting

This study was conducted in four public hospitals located in Sidama Regional State, Ethiopia, that were general and comprehensive specialized public hospitals within the regional state. The regional state has a total of 18 public hospitals, one comprehensive specialized public hospital (Hawassa University Comprehensive Specialized Hospital), four general hospitals (Adare General Hospital, Bona General Hospital, Leku General Hospital, and Yirgalem General Hospital), thirteen primary hospitals, and one hundred and thirty-three health centers [18].

Based on each hospital monthly report, which was found from Regional Health Bureau and each hospital registration, the six-month (March to August 2021) report preceding the survey showed that on average, 352, 285, and 236 women were admitted every month for emergency obstetric care in Hawassa University Comprehensive Specialized Hospital, Adare General Hospital, and Yirgalem General Hospital, respectively.

### 2.2. Study Design and Period

An institution-based, cross-sectional study was conducted in general and comprehensive specialized public hospitals in Sidama Regional State from September 13 to November 13, 2021.

### 2.3. Source Population

All women who attended emergency obstetric care in public hospitals in Sidama Regional State were the source population.

### 2.4. Study Population

All women who were admitted for emergency obstetric care in selected general and comprehensive specialized public hospitals in Sidama Regional State during the study period were the study population.

### 2.5. Eligibility Criteria

#### 2.5.1. Inclusion Criteria

All women who were admitted for emergency obstetric care in the selected hospitals and those women who were volunteered to participate in the study were included in the study.

#### 2.5.2. Exclusion Criteria

Women who were readmitted after an earlier inclusion and those who were transferred to another facility after earlier inclusion were excluded from the study. Women who were unable to provide a response due to ill health status were also excluded from the study.

### 2.6. Sample Size Determination and Sampling Procedure

#### 2.6.1. Sample Size Determination

The sample size of this study was calculated for both the magnitude and associated factors. However, larger sample was identified using the single population proportion formula considering the following assumptions: 95% confidence interval (CI), prevalence (*p*) of third delay 34.7% [19], the margin of error (*d*) 0.05, design effect 1.5, and adding 10% nonresponse rate; the final required sample size was 574.

#### 2.6.2. Sampling Techniques and Procedures

Multistage sampling technique was used to draw participants from the study population. First, by using a simple random sampling method, two public general hospitals (Adare General Hospital and Yirgalem General Hospital) were taken from all four general hospitals (Adare General Hospital, Bona General Hospital, Leku General Hospital, and Yirgalem General Hospital) and Hawassa University Comprehensive Specialized Hospital was taken directly since there was no other comprehensive specialized hospital in the regional state.

Study participants were selected from the study population by systematic sampling, in which samples were withdrawn every *K* of admitted women on discharge. After calculating *K* (*N*/*n*), where “*N*” is the total number of women expected to be discharged from the hospital after they got emergency obstetric care in the study area during the study period (two months) based on the previous 6-month statistics and “*n*” is the calculated sample size; study participants were selected at every *K*^th^ interval (1746/574 = 3.04). Therefore, samples were taken every 3^rd^ interval. The first participant was selected by lottery method.

### 2.7. Operational Definition

#### 2.7.1. Maternal Third Delay

A woman was considered as she had a maternal third delay if the time elapsed between arrival to the hospital and receiving care takes 60 minutes or more [20].

#### 2.7.2. Well Prepared for Birth and Its Complications

A mother was considered well prepared for birth and its complications if she reported completing at least two of the listed five actions which included saving money, identifying health professionals, identifying place of delivery, identifying mode of transportation, and identifying blood donor in case of emergency [21].

#### 2.7.3. Knowledgeable on Key Danger Signs of Pregnancy, Labor and Delivery, and Postpartum Period

A woman was considered as knowledgeable on key danger signs of pregnancy, labor and delivery, and postpartum period as she mentioned at least three key danger signs for pregnancy, labor and delivery, and postpartum period spontaneously [22].

#### 2.7.4. Admitted for Emergency Obstetric Care

A woman was considered as she was admitted for emergency obstetric care when she was admitted during her pregnancy, labor and delivery, or postpartum period for emergency obstetric care which includes assisted vaginal delivery; removal of retained products of conception; manual removal of the placenta; parenteral administration of oxytocin, antibiotics, and anticonvulsants; blood transfusion; and caesarean section (surgery) [23, 24].

#### 2.7.5. Absence of Health Professionals

A woman was considered as she experienced an absence of health professionals when she reported that she did not get health professionals when she needed them during her stay in the current hospital admission [20].

### 2.8. Data Collection Tools and Procedures

Data collection tool was adopted and modified after reviewing different literatures [20, 25]. The referral paper, triage paper, and patient chart were reviewed about the woman's condition. Interview regarding sociodemographic characteristics, the reason for delay (if any), and her experience in the hospital was done using a structured questionnaire in the inpatient wards every day at the time of discharge.

### 2.9. Data Quality Assurance

Data quality was assured by developing a structured questionnaire in English based on the objectives to be addressed after reviewing the relevant literature, and it was reviewed by the principal and coadvisors. The translation of the questionnaire from English to Amharic and Sidaamu Afu (the local language) was done by a language expert. The data collection was done following two days of training for data collectors and supervisors (all were BSc Midwives) after the pretest was conducted with 5% of the calculated sample size at Shashemene General Hospital. Any ambiguous and unsuitable questions were modified after checking of questionnaire completeness at pretest. The collected data were checked by supervisors and principal investigators for its completeness.

### 2.10. Data Processing and Analysis

The collected data were coded and entered into EpiData version 4.6.0.6 and exported to Statistical Package for Social Science (SPSS) version 25 for analysis. The data was cleaned to check for consistency and missing values. Descriptive statistics like mean, standard deviation, frequency and percentages, and text were used. Then, bivariable and multivariable logistic regression analyses were done. Variables with *p* value < 0.25 on bivariable analysis were adjusted for the outcome variable to identify independently associated variables. In the multivariable analysis, variables having a *p* value < 0.05 were taken as significant predictors. The model fitness was ascertained with the Hosmer and Lemeshow test, and multicollinearity test was done by using correlation matrix and variant inflation factor. Crude and adjusted odds ratios with their 95% confidence level were computed to show the association between dependent and independent variables.

### 2.11. Ethical Considerations

Approval to carry out this study was obtained from the Institutional Review Board (IRB) of Hawassa University, College of Medicine and Health Science (Ref. No: IRB/270/13). Additionally, a supporting letter from the midwifery department and informed written consent were obtained from each study participant prior to enrollment. Confidentiality was assured to the study participants throughout the study.

## 3. Result

### 3.1. Sociodemographic Characteristics of the Study Participants

Five hundred forty-two respondents participated in the study giving a response rate of 94.4%. Three hundred eighteen (58.7%) of the respondents were from urban, and 224 (41.3%) were rural residents. About two-thirds (67.3%) of the study participants were in the age group 20-29 years. The mean (SD) age was 27.06 (±4.31) years, with a range of 18-40 years. Around 15% of the respondents, 81 (14.9%) did not attend formal education and more than half (53.9%) were housewives (Table 1).

### 3.2. Obstetric Characteristics of the Respondents

A total of 361 (66.6%) of the study participants were found to have 2-4 pregnancies; 42 (7.7%) had a history of abortion; and 99 (18.3%) of them had a history of obstetric complications (Table 2).

### 3.3. Respondent's Characteristics Related to Obstetric Danger Sign Knowledge and Birth Preparedness and Complication Readiness Practice

From the total 542 respondents, 300 (55.4%), 313 (57.7%), and 232 (42.8%) were identified as knowledgeable on danger signs during pregnancy, labor and delivery, and postpartum period, respectively. In addition, 333 (61.4%) of the study participants were well prepared for birth and its complications (Table 3).

### 3.4. Admission and Hospital Stay-Related Characteristics of the Respondents

Three hundred one (55.5%) of the respondents arrived to the hospital during the day time, and 391 (72.1%) were during working days. Concerning the availability of health professional, 158 (29.2%) respondents reported that they did not get a health care provider as they need (Table 4).

### 3.5. Magnitude of Maternal Third Delay

In this study, maternal third delay was identified among 29.3% (159) (95%CI = 25.2 − 33.5) of the study participants. Time to receive emergency obstetric care ranges from less than a minute to 6 hours, and the median time to receive care was 40 minutes with an interquartile range between 25 and 60 minutes. For descriptive purposes, the time to receive emergency obstetric care was separated into three phases. The first was the time gap between arrival to the health facility and triage, which was ranged from less than a minute to 1 hour and 30 minutes, and the median time was 10 minutes. The second phase was between triage and the first examination by a health professional, and it ranges from less than a minute to 1 hour and 20 minutes with a median of 6 minutes. The third phase, which was described as the gap between the first examination by a health professional and receiving care, ranges from less than a minute to 5 hours and 45 minutes, and the median was 15 minutes.

### 3.6. Reason for Maternal Third Delay as Described by the Respondents

Based on women's reports, the top identified reason for maternal third delay was staff workload (47.2%). Additionally, long admission process 35 (22.0%), health professional negligence 27 (17%), and a problem with supply or medication 23 (14.5%) were also identified as the reasons for their delay in receiving care (Table 5).

### 3.7. Factors Associated with Maternal Third Delay

In the results of bivariable logistic regression analysis, maternal third delay was associated with women's educational status; women's occupational status; decision-maker for obstetric care; knowledge on danger signs of pregnancy, labor and delivery, and postpartum; referral status of the women; ANC visit; BPCR practice; absence of health professional; lengthy admission process; date of admission; pregnancy status on admission; mode of termination of pregnancy; and experiencing obstetric complication with significance level of *p* < 0.25 (Table 6).

On the multivariable logistic regression, women's occupational status, referral status of the women, ANC visit, BPCR practice, and absence of health professionals were retained associated with maternal third delay (*p* value < 0.05) Compared to housewives, self-employees and government employees have 77.7% (AOR = 0.223, 95%CI = 0.122 − 0.409) and 84.3% (AOR = 0.157, 95%CI = 0.063 − 0.396) less likely to face maternal third delay, respectively. Women who have no ANC follow-up were three times (AOR = 2.795, 95%CI = 1.318 − 5.928) more likely to face maternal third delay than those who have ANC follow-up. Related to birth preparedness and complication readiness practices, women who were not well prepared had two times (AOR = 2.418, 95%CI = 1.51 − 3.869) more likely to face maternal third delay.

Women who experienced referral from health facilities were almost 68.9% (AOR = 0.311, 95%CI = 0.181 − 0.534) less likely to encounter maternal third delay compared to those women who get the service in their first contact visit. Similarly, women who did not find health professionals as they needed were five times (AOR = 4.63, 95%CI = 2.857 − 7.50) more likely to have maternal third delay compared to their counterparts.

## 4. Discussion

This institution-based study has attempted to identify the magnitude and associated factors of maternal third delay in public general and comprehensive specialized hospitals in Sidama Regional State, Southern Ethiopia. The result showed that maternal third delay among women admitted for emergency obstetric care in public general and comprehensive specialized hospitals in Sidama Regional State was 29.3% (95%CI = 25.2 − 33.5), and it is in line with the study done in Gamo Zone (31.7%) [20], Arsi Zone (25.5) [26], Nigist Eleni Mohammed Memorial General Hospital (32.6%) [15], Bahir Dar City (30.7%) [27], three referral hospitals of Amhara Regional State (26.9%) [16], and Yem Special Woreda (34.7%) [19]. This might be for having similar health policy and strategy.

However, in this study, the magnitude of maternal third delay is lower than the study done in two referral hospitals in Addis Ababa (74.7%) [17]. This difference might be due to variation in the study setting, sample size, population size, women's socioeconomic and demographic characteristics, fee-free services in the study area, advanced medical logistics supply, and professional staff. The two referral hospitals in Addis Ababa [17] provide services with fees, and the population size was incomparably high in contrast to the study area which may affect service provision.

In reverse, the magnitude of maternal third delay is higher than the study conducted in University Teaching Hospital of Ouagadougou (UTH-YO), Burkina Faso (8.2%) [28]. The reason might be the study was conducted in a single hospital. Additionally, the difference in health policy and having an improved maternal health profile in Burkina Faso might contribute for this result [29].

Based on women reports of their reason for experiencing delay in receiving care, staff workload 75 (47.2%), lengthy admission process 35 (22%), health professionals negligence 27 (17%), lack of supply or drugs 23 (14.5%), delay in providing informed consent 3 (1.9%) and unfunctional operating room 2(1.3%) were stated. These findings were also identified as a reason for maternal third delay in a cross-sectional study conducted in Brazil [30], University of Gondar Referral Hospital [31], Arsi Zone [26], and a multicountry study done in seven countries [10].

Women's occupational status, not receiving emergency obstetric care at the first contact visit (referred from another health facility), ANC follow-up, BPCR practice, and absence of health professionals were significant predictors of maternal third delay.

Women whose occupations were self-employed and government employees have 77.7% and 84.3% less likely to experience maternal third delay compared to housewives, respectively. This was consistent with the study done in Arsi Zone [26]. However, none of the sociodemographic variables were associated with maternal third delay in a study done in Bahir Dar City [27]. The reason for the lower maternal delay among self-employed and government employees might be these groups of women might have ANC follow-up and well prepare for birth and its complication [32]. Additionally, they might identify and report obstetric danger signs early.

Related to ANC follow-up, women who had no antenatal follow-up were three times more likely to experience a third delay compared to those who had one or more antenatal follow-up. Similar result was also found in a study done in Arsi Zone [26]. Additionally, a study conducted in West Bengal, India, also reported that women who did not attend ANC had a higher proportion of maternal third delay [33]. This might be for the reason that women who had ANC follow-up might be knowledgeable on obstetric danger signs and well prepared for birth and its complication [34]. Other possible explanations might be the experience of women to communicate with health care providers; and they might know to whom and how to communicate for their problems.

Regarding the absence of health professional, women who reported the absence of health professionals when they need them were five times more likely to have maternal third delay compared to their counterparts. This result is in line with studies done in Gamo Zone, Ethiopia, which identified that women who reported the absence of health professionals when they need them were ten times more likely to experience maternal third delay [20]. Additionally, this association was found in a study conducted in Hadiya Zone [15]. This might be for a reason that there might be a gap in the availability of health professional to give care due to staff workload and/or motivation.

Referral status of the women was another factor associated with maternal third delay. Women who experienced referral from health institutions were almost 68.9% less likely to encounter this type of delay than those women who got the service in their first contact visit. This result is in line with a study done in another study in Ethiopia which shows 80% less likely to experience this type of delay [15]. This might be for the reason that women who were referred from other health facilities might have obstetric complications for which human resources and supplies might be mobilized [26]. Additionally, the receiving facilities might be communicated before they refer the woman that might make the receiving hospital know the woman health condition and get ready to manage her health problem.

Additionally, BPCR practice was significantly associated with maternal third delay. Women who were not well prepared for birth and its complications were found to be two times more likely to experience this type of delay. This could be due to the reason that these women might solve problems related to financial issues when they encounter lack of supply or drugs and give consent timely; and they might also report obstetric danger signs early.

This study has certain limitations. Since the data were collected from women perspective and medical chart which might not be enough to describe maternal third delay in in-depth way, this could be improved with the inclusion of qualitative method and direct observational studies. Recall bias might also be a problem since women were asked about their experience. The utilization of secondary data might be a problem. Therefore, the interpretation of the findings should be made carefully.

## 5. Conclusion and Recommendation

This study identified that maternal third delay was high and several women were not receiving emergency obstetric care in the recommended time range after they arrived at the health facilities. Being government employed and self-employed, having ANC follow-up, being well prepared for birth and its complications, arriving with referral from other health facilities, and absence of health professionals were significant predictors of maternal third delay. Therefore, it is better to incorporate the identified factors (women's occupation, referral status, ANC follow-up, BPCR practice, and absence of health professionals) during the implementation and evaluation of maternal health programs to decrease maternal third delay.

## Figures and Tables

**Table 1:** Sociodemographic characteristics of the respondents admitted in general and specialized hospitals in Sidama Regional State, Ethiopia, 2021.

Variables	Categories	Frequency	Percent (%)
Age (in completed years)	15-19	20	3.7
	20-24	135	24.9
	25-29	229	42.3
	30-34	134	24.7
	≥35	24	4.4
Residence	Urban	318	58.7
	Rural	224	41.3
Marital status	Married	516	95.2
	Single	16	3
	Other^∗^	10	1.8
Educational level	No formal education	81	14.9
	Primary school	226	41.7
	Secondary school	171	31.5
	College and above	64	11.8
Occupational status	Housewives	292	53.9
	Self-employee	148	27.3
	Government employee	77	14.2
	Other^∗∗^	25	4.6
Partner educational level	No formal education	47	8.7
	Primary school	145	26.8
	Secondary school	248	45.8
	College and above	76	14.0
	Not having partner	26	4.8
Partner occupational status	Farmer	166	30.6
	Government employee	112	20.7
	Merchant	138	25.5
	Private employee	75	13.8
	Other^∗∗∗^	25	4.6
	Not having partner	26	4.8
Decision-maker at home	Woman	95	17.5
	Partner or family	140	25.8
	Jointly	307	56.6
Main source of income	Partner	440	81.2
	Women	91	16.8
	Family	11	2
Family monthly income	≤500	26	4.8
	501-1000	133	24.5
	1001-2000	148	27.3
	≥2001	235	43.4

^∗^Divorced and widowed. ^∗∗^Private employee and student. ^∗∗∗^Daily laborer.

**Table 2:** Obstetric characteristics of the respondents admitted in general and specialized hospitals in Sidama Regional State, Ethiopia, 2021.

Variables	Categories	Frequency	Percent (%)
Gravidity	1	121	22.3
	2-4	361	66.6
	≥5	60	11.1
Parity	0	16	3.0
	1-3	433	79.9
	≥4	93	17.2
History of abortion	No	500	92.3
	Yes	42	7.7
Number of children alive	0-1	18	3.3
	2-3	455	83.9
	≥4	69	12.7
History of obstetric complication	No	443	81.7
	Yes	99	18.3
Type of pregnancy	Wanted and planed	427	78.8
	Wanted but unplanned	81	14.9
	Unwanted and unplanned	34	6.3
ANC visit	No visit	57	10.5
	Had visit	485	89.5
GA on admission	<28 weeks	43	7.9
	28 to 36 weeks	60	11.1
	37 and above	412	76.0
	Postpartum or postabortion	27	5.0
Obstetric complication in the current admission	No	289	53.3
	Yes	253	46.7
Mode of termination for current pregnancy	SVD	262	48.3
	Cesarean section	157	29
	Uterine evacuation	37	6.8
	Induction/augmentation	34	6.3
	Other	20	3.7
	Not terminated	32	5.9

**Table 3:** Obstetric danger sign knowledge and birth preparedness and complication readiness practice-related characteristics of the respondents admitted to general and specialized hospitals in Sidama Regional State, Ethiopia, 2021.

Variables	Categories	Frequency	Percent (%)
Knowledge on danger sign of pregnancy	Not knowledgeable	242	44.6
	Knowledgeable	300	55.4
Knowledge on danger sign of labor and delivery	Not knowledgeable	229	42.3
	Knowledgeable	313	57.7
Knowledge on danger sign of postpartum	Not knowledgeable	310	57.2
	Knowledgeable	232	42.8
Knowledgeable on danger sign of pregnancy, labor and delivery, and postpartum	Not knowledgeable	384	70.8
	Knowledgeable	158	29.2
Birth preparedness and complication readiness practice	Not well prepared	209	38.6
	Well prepared	333	61.4

**Table 4:** Admission and hospital stay-related characteristics of the respondents admitted in general and specialized hospitals in Sidama Regional State, Ethiopia, 2021.

Variables	Categories	Frequency	Percent (%)
Time of admission	Day time	301	55.5
	Night time	241	44.5
Date of admission	Working day	391	72.1
	Calendar day	151	27.9
Referred from other health facility	No	334	61.6
	Yes	208	38.4
Source of referral	Health center	139	25.6
	Government hospital	49	9.0
	Private hospital	17	3.1
	Private clinic	3	.6
	Non referral cases	334	61.6
Absence of health professional	No	384	70.8
	Yes	158	29.2
Lack of medical supply/medication	No	474	87.5
	Yes	68	12.5
Lengthy admission process	No	443	81.7
	Yes	99	18.3

**Table 5:** Reason for maternal third delay as described by the respondents admitted in general and specialized hospitals in Ethiopia, 2021.

Reasons	Frequency	Percent (%)
Staff workload	75	47.2
Health professionals negligence	27	17.0
Lack of supply or medication	23	14.5
Long admission process	35	22.0
Delay in providing informed consent	3	1.9
Unfunctional OR	2	1.3
Other^∗^	6	3.8
Reason not mentioned	28	17.6

^∗^Laboratory result delay, waiting for family member, and not identifying the ward.

**Table 6:** Bivariable and multivariable logistic regression for factors associated with maternal third delay among respondents in general and specialized hospitals in Sidama Regional State, Ethiopia, 2021.

Variables	Maternal third delay (*n*, %)		Unadjusted OR (95% CI)	Adjusted OR (95% CI)
	No delay	Delayed		
Women's occupational status				
House wife	170 (58.2)	122 (41.8)	1	1
Self-employee	126 (85.1)	22 (14.9)	0.243 (0.146-0.405)	0.223 (0.122-0.409)^∗∗^
Government employee	69 (89.6)	8 (10.4)	0.162 (0.075-348)	0.157 (0.063-0.396)^∗∗^
Other	18 (72)	7 (28)	0.542 (0.220-1.337)	0.478 (0.160-1.426)
Referred from other health facility				
Not referral	220 (65.9)	114 (34.1)	1	1
Referral	163 (78.4)	45 (21.6)	0.533 (0.357-0.795)	0.311 (0.181-0.534)^∗∗^
ANC visit				
No visit	25 (43.9)	32 (56.1)	3.608 (2.059-6.32)	2.795 (1.318-5.928)^∗^
Had visits	358 (73.8)	127 (26.2)	1	1
BPCR practice				
Not well prepared	119 (56.9)	90 (43.1)	2.894 (1.98-4.236)	2.418 (1.51-3.869)^∗∗^
Well prepared	264 (79.3)	69 (20.7)	1	1
Absence of health professional				
No	311 (81.0)	73 (19.0)	1	1
Yes	72 (45.6)	86 (54.4)	5.089 (3.398-7.620)	4.63 (2.857-7.50)^∗∗^
Lengthy admission process				
No	318 (71.8)	125 (28.2)	1	1
Yes	65 (65.7)	34 (34.3)	1.331 (.837-2.115)	1.753 (.988-3.109)
Day of admission				
Working day	262 (67)	129 (33)	1	1
Calendar day	121 (80.1)	30 (19.9)	0.504 (0.32-0.791)	0.725 (0.425-1.239)
Currently faced obstetric complication				
No	197 (68.2)	92 (31.8)	1	1
Yes	186 (73.5)	67 (26.5)	0.771 (0.531-1.120)	0.804 (0.400-1.616)
Woman's educational status				
No formal	41 (50.6)	40 (49.4)	5.268 (2.360-11.762)	1.700 (.635-4.554)
Primary	157 (69.5)	69 (30.5)	2.373 (1.142-4.933)	1.119 (.452-2.772)
Secondary	131 (76.6)	40 (23.4)	1.649 (0.770-3.533)	1.149 (.459-2.875)
College and above	54 (84.4)	10 (15.6)	1	1

OR: odds ratio; CI: confidence interval; ^∗^statistically significant at *p* value < 0.05; ^∗∗^statistically significant at *p* value < 0.001; 1: referent variable.

## Data Availability

The data sets analyzed during this study are not available publicly due to privacy reasons but are available from the corresponding author on request with rational reason.

## Abbreviations

ANC: Antenatal care
AOR: Adjusted odds ratio
BPCR: Birth preparedness and complication readiness
CI: Confidence interval
COR: Crude odds ratio
C/S: Cesarean section
EDHS: Ethiopian demographic health survey
ICU: Intensive care unit
MMR: Maternal mortality ratio
SPSS: Statistical Package for Social Science
SDG: Sustainable Development Goals
WHO: World Health Organization.
